# Physicomechanical and Morphological Characterization of Multi-Structured Potassium-Acrylate-Based Hydrogels

**DOI:** 10.3390/gels8100627

**Published:** 2022-10-01

**Authors:** José Luis Gradilla-Orozco, José Ángel Hernández-Jiménez, Oscar Robles-Vásquez, Jorge Alberto Cortes-Ortega, Maite Renteria-Urquiza, María Guadalupe Lomelí-Ramírez, José Guillermo Torres Rendón, Rosa María Jiménez-Amezcua, Salvador García-Enriquez

**Affiliations:** 1 Department of Engineering, Centre for Industrial Technical Education, Guadalajara 44630, Mexico; 2 Department of Wood Cellulose and Paper, University of Guadalajara, Guadalajara 44430, Mexico; 3 Department of Chemical Engineering, University of Guadalajara, Guadalajara 44430, Mexico; 4 Department of Chemistry, University of Guadalajara, Guadalajara 44430, Mexico

**Keywords:** hydrogels, photo-polymerization, potassium acrylate-co-acrylamide, swelling capacity, multistructured

## Abstract

In this work, a photo-polymerization route was used to obtain potassium acrylate-co-acrylamide hydrogels with enhanced mechanical properties, well-defined microstructures in the dry state, and unique meso- and macrostructures in the hydrated state. The properties of the hydrogels depended on the concentration of the crosslinking agent. Mechanical properties, swelling capacity, and morphology were analyzed, showing a well-defined transition at a critical concentration of the crosslinker. In terms of morphology, shape-evolving surface patterns appeared at different scales during swelling. These surface structures had a noticeable influence on the mechanical properties. Hydrogels with structures exhibited better mechanical properties compared to unstructured hydrogels. The critical crosslinking concentration reported in this work (using glycerol diacrylate) is a reference point for the future preparation of multistructured acrylic hydrogel with enhanced properties.

## 1. Introduction

Hydrogels are polymeric tridimensional networks that can absorb large amounts of water and other fluids without compromising their structure [1,2,3]. Their swelling depends on the presence of certain functional groups, crosslinking degree, chain flexibility, tacticity, crystallinity of components [4], and thermal history [5]. These unique materials are being used in several commercial applications, for example, as ophthalmic devices, biosensors, biological membranes, and drug carriers [1,2]. Potential applications of hydrogels as soil conditioners and as removal agents for heavy metal ions have also been mentioned in the literature [6,7,8]. The properties of hydrogels strongly depend on the synthesis, concentration, and nature of components and the polymerization process. In this regard, several studies dealing with methods of synthesis, effects of swelling, and crosslinkers on properties have been reported [9,10,11,12,13,14,15,16,17,18,19,20,21,22].

UV curing (photopolymerization) is an alternative process for synthesis of hydrogels. It allows for a better control over the reaction kinetics and it is not affected by the presence of oxygen in the system [23,24,25]. Additionally, photopolymerization can be utilized with most monomers, and only one additive (photoinitiator) is needed [26,27]. Other advantages of photopolymerization are short times of synthesis and minimum generation of heat [28]. It is also important to mention that photopolymerization is widely used to prepare hydrogels with applicability in medical and biological fields. For example, successful cell encapsulation and high cell viability in photopolymerized hydrogels [23] and the development of advanced photoinitiators suitable for several medical conditions [29] have been reported. Moreover, excellent resistance against bacteria in photopolymerizable hydrogels have also been reported. Lin et al. (2011) prepared a biocompatible silicone based on carboxybetaine and a macromer (bis-α, ω-(methacryloxypropyl) poly-dimethylsiloxane) via photopolymerization using Darocur^®^ TPO as the photo-initiator. These hydrogels showed excellent resistance against bacterial adhesion and protein adsorption [30]. 

In the case of acrylic hydrogels prepared via photopolymerization, there are also plenty of published studies, from copolymer systems [31] to reinforced hydrogels [22,32,33], among others. In the case of photopolymerized hydrogels based on potassium polyacrylates, there are only a few studies reported. Ruan et al. (2004) studied polyacrylate potassium and polyacrylate sodium hydrogels using different photoinitiators in the synthesis. They found that the highest water absorptions were exhibited by hydrogels synthesized with Irgacure 1700 and Irgacure 1800 [34]. In this work, we utilized Irgacure 1700. 

**Figure 1:**
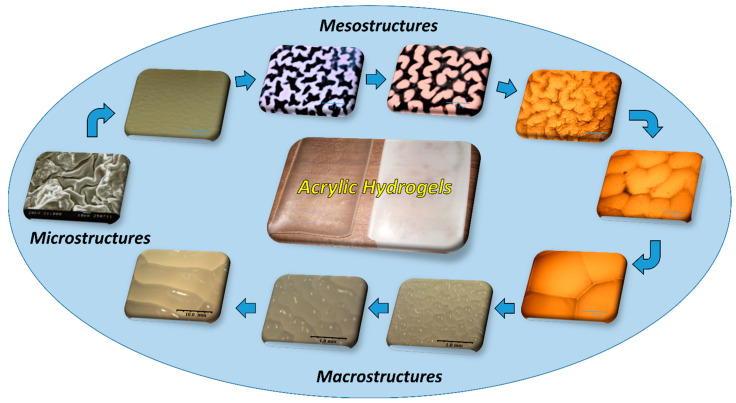
Surface patterns of acrylic hydrogels.

The superficial instabilities that appear during swelling in gels are a phenomenon known since the XIX century [35]. Tanaka et al. (1987) observed the appearance of patterns in polyacrylamide-based gels during phase transition. This phenomenon affected the understanding of the kinetic process in the gels [36]. In another study by Tanaka et al. (1992), the morphologic evolution and kinetics of superficial patterns in acrylic gels during swelling was reported. A dynamic ordering of patterns was observed [37]. Li et al. (1994) reported the presence of hexagonal-, grain-, and bubble-like shape patterns in ionic N-isopropylacrylamide (N-IPA)-based gels. These patterns were observed below, near, and above the transition phase temperature, and their behavior depended on the temperature, time, external constraint, and thermal history [38]. In a study dealing with photopolymerizable polyhydroxythylmethacrylate (PHEMA) hydrogel films with a crosslinking gradient, Guvendiren et al. (2009) reported a method that allowed them to form various osmotically driven surface patterns without organic solvents for swelling. They observed and captured the shape evolution of such patterns, being first hexagonal structures, then peanut shapes, and then lamellar and finally worm-like patterns [39]. The same research group later reported creasing formation in the gradient PHEMA hydrogels using various solvents. They found that the morphology of patterns depended on the equilibrium linear expansion, which was as a function of the solvent–polymer interaction and the concentration of the crosslinker [40]. Recently, Chuang et al. (2021) demonstrated that the UV irradiation dose and the immersion conditions in DI water determined the characteristics of surface patterns in pHEMA-based hydrogels [41]. The maximum characteristic wavelength of the formed wrinkles depended on the initial immersion time. This dependency had a relationship that followed the power law. Furthermore, it is important to mention that surface structures seem to have an important effect on cells attached on hydrogels. In this regard, Saha et al. (2010) reported that wrinkled patterns on the surface of soft hydrogels made of polyacrylamide greatly influenced cell attachment and cell behavior [42]. Figure 1 displays some of the surface patterns that can be generated in acrylic hydrogels. 

It is clear that there is a great interest in polymeric systems that display spontaneous formation of patterns that can be controlled in terms of size, order, morphology, and complexity. Such materials could be useful in many applications such as coatings, optical filters, batteries, actuators, valves, microfluidic devices, and flexible electronics [43,44,45,46,47]. In this work, we prepared multi-structured hydrogels of poly(potassium acrylate-co-acrylamide) via photopolymerization. Photo initiation allowed us to control the temperature of the synthesis, which led a higher structural stability of hydrogels during swelling. We used the term multi-structured due to the ability of these hydrogels to display patterns/structures at different scales (micro-, meso-, and macroscales). The effect of the crosslinking agent (glycerol diacrylate or DAG) on the swelling capacity, morphology, and mechanical properties was evaluated.

## 2. Results and Discussion

**Figure 2:**
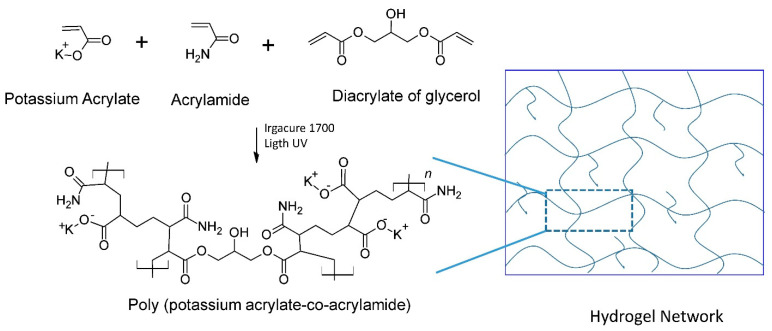
Proposed formation of the polymeric network.

Figure 2 shows the probable reaction between the acrylic monomers and the crosslinking agent to form the polymeric network. Undesired residues such as unreacted molecules from the pho-initiator and the monomers, as well as free oligomers, were removed in the cleaning step [48].

During preparation of hydrogels, it was possible to control the temperature of the reaction by means of photo-polymerization. By controlling the entropy of the reaction solution at low temperatures, an ordered polymeric network was formed, allowing for the preparation of transparent and homogeneous hydrogels. These hydrogels presented a fractal-like structure in three different scales: micro (10^−7^ to 10^−8^ m), meso (10^−5^ to 10^−7^ m), and macro (10^−3^ to 10^−5^ m). Such properties were consistent with those from hydrogels synthetized via redox initiation [20]. The polymer gel fraction (*G_F_*) was calculated for all hydrogels (shown in Table 1), as mentioned in the experimental section. All values were higher than 91%, which is consistent with values reported by Rodgers et al., who determined *G_F_*’s of 89% and 90% for acrylamide and 93% for acrylic acid [29]. Lara-Valencia et al. [10] reported values lower than 90% for acrylic hydrogels obtained by redox initiation and higher than 90% for photoinitiated ones. Wen et al. [31] reported values lower than ours for poly acrylic acid/cellulose nanofibers hydrogels. This could be attributed to the nanofibers, which could have had decreased the UV light transmittance. 

### 2.1. Infrared Spectroscopy FTIR

**Figure 3:**
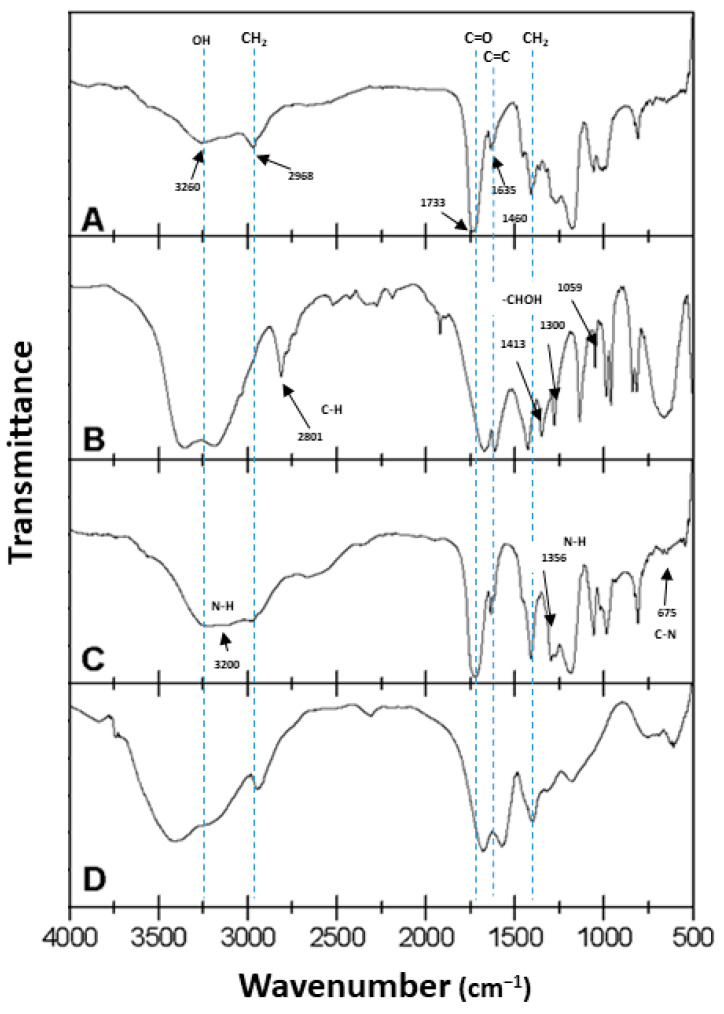
FTIR spectra for (**A**) acrylic acid, (**B**) DAG, (**C**) acrylamide, and (**D**) hydrogel with 4 wt % DAG.

Figure 3A shows the FTIR spectrum corresponding to acrylic acid. Here, the bands at 3260 cm^−1^ were attributed to the OH groups, while the bands corresponding to the asymmetric and symmetric tension vibrations of CH_2_ were detected at 2968 cm^−1^ and 1460 cm^−1^, respectively. The band at 1733 cm^−1^ corresponded to the carboxylic carbonyl group, while the one at 1635 cm^−1^ was attributed to the vibration of the C=C double bond. The out-of-plane deformation (=C-H) was represented by the band at 812 cm^−1^. Figure 3B corresponds to the FTIR spectrum of the glycerol diacrylate (DAG), in which the same characteristic bands were observed as in the spectrum shown in Figure 3A. This was because both compounds possess the same functional groups. However, here, we had the presence of a secondary alcohol (-CHOH) that generated bands at 1059 cm^−1^, 1300 cm^−1^, and 1413 cm^−1^. Figure 3C shows the FTIR spectrum of acrylamide. Here, we had the combination of N-H strain and C-N bending in the bands at 1356 cm^−1^ and 675 cm^−1^, respectively, while the combination of N-H strain and C-N strain occurred at 1610 cm^−1^. Bands corresponding to the double bond C=O (≈1735 cm^−1^) and the N-H strain (from 3342 to 3200 cm^−1^) were also observed. It can be noted that the band for the carbonyl group (C=O) appeared at 1670 cm^−1^, which could be attributed to the presence of the amino group [10]. In Figure 3D, the spectrum obtained for the acrylamide/potassium acrylate copolymer, with 4 wt % glycerol diacrylate (DAG), is shown. Here, the absence of the C=C double bond signal was evident, and the presence of the characteristic signals for both monomers can be noted. Bands located at 1677 and 1572 cm^−1^ can be attributed to the carbonyl and amino groups, respectively.

### 2.2. Swelling Kinetics

**Figure 4:**
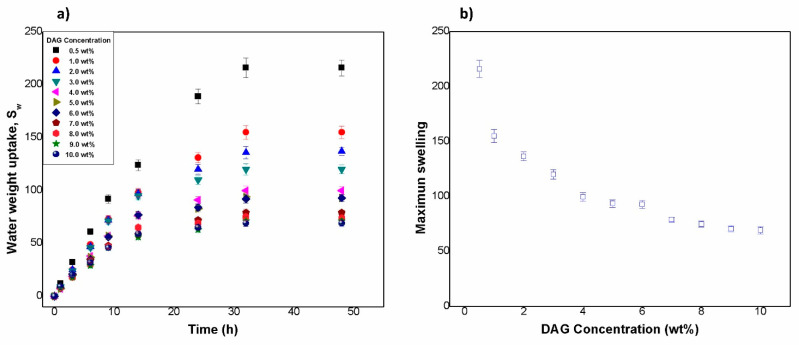
(**a**) Water weight uptakes of hydrogels as a function of time. (**b**) Maximum swelling as a function of the crosslinking agent (DAG).

Figure 4a shows the swelling kinetics of the obtained hydrogels. The swelling of hydrogels with 0.5 wt % DAG was significantly higher than the rest. On the other hand, hydrogels with DAG concentrations from 1 wt % to 10 wt % showed a very similar behavior, having their swelling capacities reduced with increasing crosslinking. It can also be observed that groups of samples followed practically the same behavior (groups of 1, 2, and 3 wt %; groups of 4, 5, and 6 wt %; and groups of 7, 8, and 9 wt %). The maximum swelling ranged from 69 to 217 times the weight of the xerogel. This behavior depended on the amount of DAG (Figure 4). Table 1 shows the values of the maximum swelling for all formulations. Magalhães et al. [49] reported swelling degree values from 144 to 189 for hydrogels where they varied the amount of sodium acrylate in the formulation of poly(sodium acrylate-co-acrylamide) hydrogels. Leitão et al. [50] reported swelling degrees from 620 to 1100 times in acrylamide/potassium acrylate hydrogels crosslinked at 0.05, 0.1, and 0.2% mol with respect to the total mass of monomers.

Figure 4b shows the maximum swelling as a function of the crosslinking agent (DAG). A change in slope was observed at 4 wt % of DAG, similar to the characteristic length (λ) of the mesostructures and macrostructures at different times (as can be seen in the figure of the characteristic lengths). This critical value of 4 wt % of DAG is consistent with a critical crosslinking concentration reported in a study from Lopez-Ureta et al. (2008), in which the authors synthetized acrylic acid/acrylamide hydrogels via redox initiation. They also used DAG as a crosslinker [20]. We believe that, starting from this critical concentration (4 wt %), stronger networks were formed, which led to less swelling in the hydrogels. At lower DAG concentrations, the hydrogels physically collapsed near the equilibrium, which can be attributed to weaker networks.

**Figure 5:**
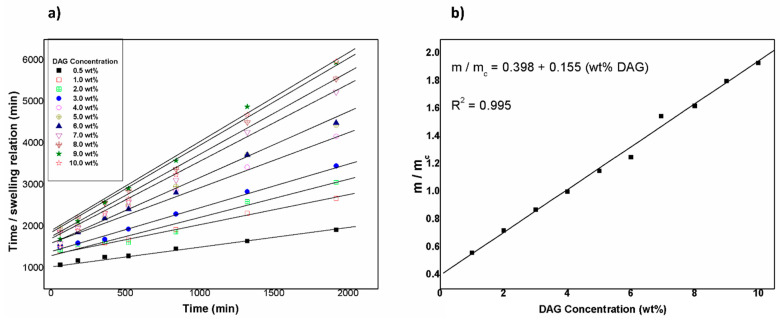
(**a**) Linearization of the Schott model for all samples. (**b**) Normalized slopes from the linearization of the Schott model (m/m_c_) as a function of DAG concentration.

The Schott’s model was used in order to analyze the adsorption behavior of hydrogels in water. Figure 5a shows the linear behavior obtained from Equation (5) (see below in the Experimental section), which showed linearity values close to 1, demonstrating that it followed the model adequately. 

In this context, Figure 5b shows the normalized slopes for all samples (m/m_critical_). Here, a linear dependence can be observed. Table 1 shows the values of the constant K for all the samples evaluated.

### 2.3. Surface Analysis

**Figure 6:**
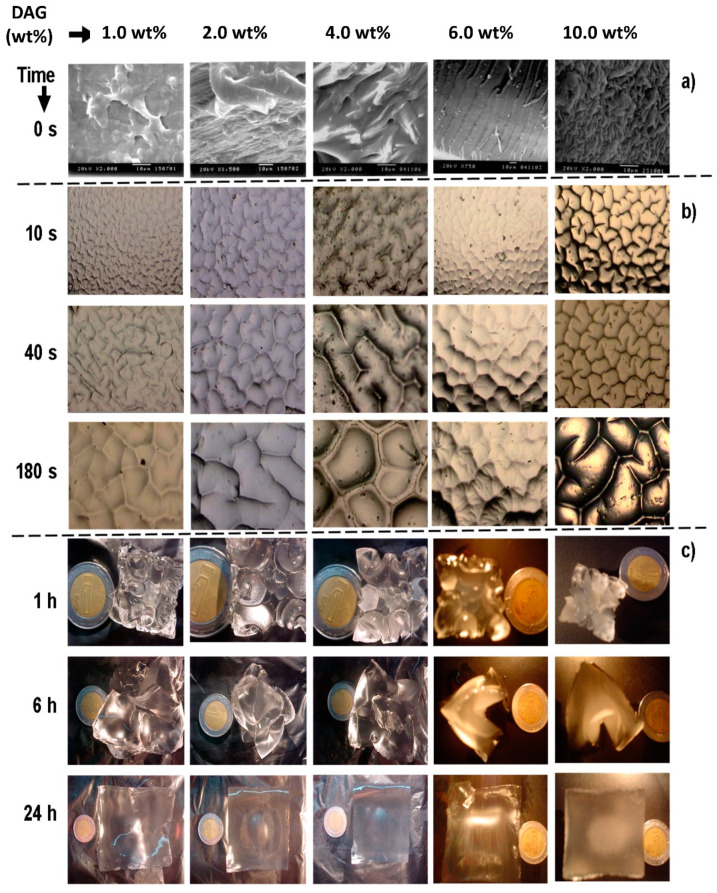
Images for hydrogels with 1 wt %, 2 wt %, 4 wt %, 6 wt %, and 10 wt % of DAG. (**a**) Micrograph (SEM) of xerogels; (**b**) polarized light micrographs of swollen hydrogels; (**c**) macrostructures of swollen hydrogels; all as functions of swelling time.

Figure 6a shows images of the surface of the xerogels where it can be observed that with a higher the amount of DAG, the structuring of the hydrogel was more noticeable. A critical crosslinking concentration (CCLC) of around 4 wt % was observed. For DAG concentrations below 4 wt %, an irregular surface arrangement was noted, while for DAG concentrations higher than 4 wt %, a compact surface, aligned preferentially in one direction, and with a more regular surface texture, was observed. Similar results were reported by López-Ureta et al. [20]. The characteristic length of the structure was in the microscale in xerogels with 10 wt % of DAG but increased when the concentration of DAG decreased. The mesostructure of hydrogels was observed by optical microscopy. When the water penetrated the hydrogels, four distinctive wrinkle patterns were spontaneously formed and transited to random worms, lamellae, peanuts, and ordered hexagonal patterns during swelling, until the maximum swelling was reached. Figure 6b shows images of hydrogels with 1 wt %, 2 wt %, 4 wt %, 6 wt %, and 10 wt % of DAG at short hydration times (10, 40, and 180 s). The order of magnitude of the structures was in the range of 10^−6^ m. The size of structures increased with hydration time, becoming more defined as the amount of DAG increased. There was evidence in this research that the crosslinking gradient plays a critical role in the evolution of surface patterns and their ordering along with the lateral confinement of the hydrogel, promoting anisotropic osmotic pressure along with the thickness. Similar systems were described [35,36,39,40].

Figure 5b shows images of hydrogels exhibiting meso- and macrostructures at different swelling times as a function of DGA. The macrostructure was defined from the first minutes of contact of the xerogel with water. After 24 h, all macrostructures disappeared. Equilibrium was reached approximately 32 h after the start of the swelling process. Depending on the degree of crosslinking of the hydrogel, the hydrogel can undergo large volume changes during swelling. During the water absorption process, the resulting compressive stresses can be quite large, even exceeding the elastic modulus of the gel. When this compressive stress becomes large enough (and the material cannot delaminate and buckle macroscopically), an elastic instability arises in which the free surface folds in on itself to locally relieve the compressive stress.

Table 1 shows the average characteristic length values for the mesostructure, measured at 60 s of hydration. Initially, the gel was in an almost stress-free state; however, immersion in a solvent led to swelling of the network until the osmotic stress, due to the mixing of solvent with polymer chains and counterions, was balanced by the elastic strain due to chain stretching. Because of the mechanical constraint provided by the substrate, a gel attached to the surface that was much thinner than its lateral dimensions can only expand in the direction normal to the surface. The result of this uniaxial expansion is that the gel undergoes a state of equibiaxial compressive stress [35].

**Figure 7:**
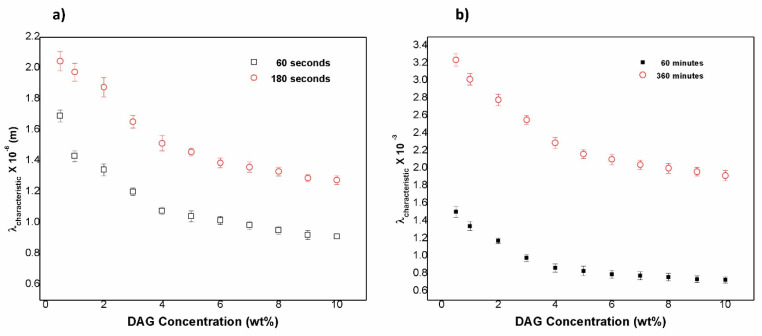
Characteristic length (λ) of structures at (**a**) 60 s and 180 s and at (**b**) 60 min and 360 min as a function of the DAG concentration.

Figure 7a shows the characteristic length (λ) of the mesostructures at 60 s and 180 s as a function of DAG concentration. The order of magnitude (10^−6^ m) increased with the water absorption. Similar to Figure 3B and Figure 7b, when the DAG concentration was 4 wt %, there was a clear change in the behavior of the data. This was probable due to the reordering of the polymeric network. Figure 7b displays the relationship between λ and the DAG concentration, but this time at 60 and 360 min. The order of magnitude was 10^−3^ m. The mesoestructures disappeared when the hydrogels reached their swelling equilibrium.

The size of the meso- and macrostructures slightly decreased when the DAG concentration also decreased, generating regular “packed” shapes and thus more compacted hydrogels. This type of superficial morphology was observed in all sides of the samples (cubes), demonstrating the tridimensional nature of the patterns. The size of these well-defined structures augmented with the time after immersion. However, the number of these structures remained constant.

### 2.4. Mechanical Tests

**Figure 8:**
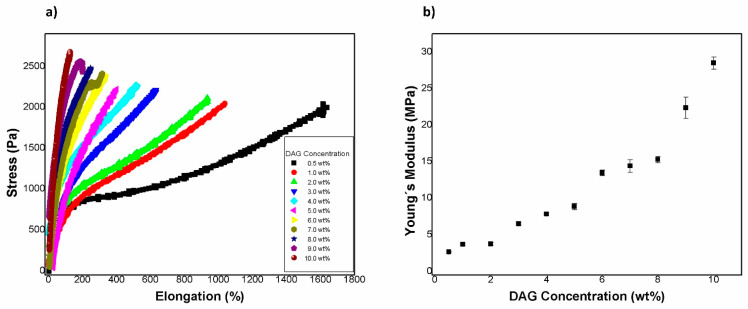
(**a**) Tensile test curves for all compositions evaluated. (**b**) Average Young’s modulus as a function of the concentration of the crosslinking agent (DAG).

Table 2 shows the values of tensile strength, elongation at break, and Young’s modulus. Figure 8a displays tensile test curves for all compositions evaluated, and Figure 8b shows the Young’s modulus as a function of the concentration of DAG. It can be observed that the elastic modulus increased when the concentration of DAG increased. In the case of the elongation at break, it was observed that it decreased when DAG concentration increased. For the tensile strength, it generally increased when the concentration of DAG increased as well. These results were expected due to the formation of stronger and stiffer polymeric networks as the DAG concentration increased.

## 3. Conclusions

By means of the photo-polymerization of potassium acrylate and acrylamide, the temperature of the synthesis was controlled. This allowed for the preparation of transparent and homogeneous hydrogels based on potassium acrylate-co-acrylamide that were obtained via photo-polymerization. The resulting hydrogels presented a fractal-like structure in the micro-, meso-, and macroscales. The maximum swelling ranged from 69 to 217 times the weight of the xerogel. This swelling behavior depended on the amount of the crosslinking agent (DAG). The critical concentration of DAG was 4 wt %, which could be a reference point to produce a hydrogel with better mechanical properties and structure characteristics. Upon increasing the amount of DAG, the mechanical resistance increased, and simultaneously, values of elongation at break and swelling capacity decreased. The morphology, swelling capacity, and Young’s modulus showed a transition between 4 and 5 wt % of DAG. This work is a starting point for the future preparation of advanced multi-structured hydrogel materials that could have a wide range of applications, such as coatings, batteries, flexible electronics, actuators, and optical filters.

## 4. Materials and Methods 

### 4.1. Materials

The monomers used for hydrogel preparation were acrylamide and acrylic acid from Aldrich with purities of 98.5% and 99.3%, respectively. Potassium hydroxide (KOH), 99%, was also purchased from Aldrich. As a photoinitiator, a mixture of 25% bis(2,6-dimethoxybenzoyl)-2,4,4-trimethyl pentylphosphineoxide and 75% 2-hydroxy-2-methyl-1-phenyl-propane-1-one (Irgacure 1700) from Ciba Speciality Chemicals Inc. was used. Glycerol diacrylate (DAG), 97% purity, was obtained from Industria Azteca Integral.

### 4.2. Synthesis of Hydrogels

**Figure 9:**
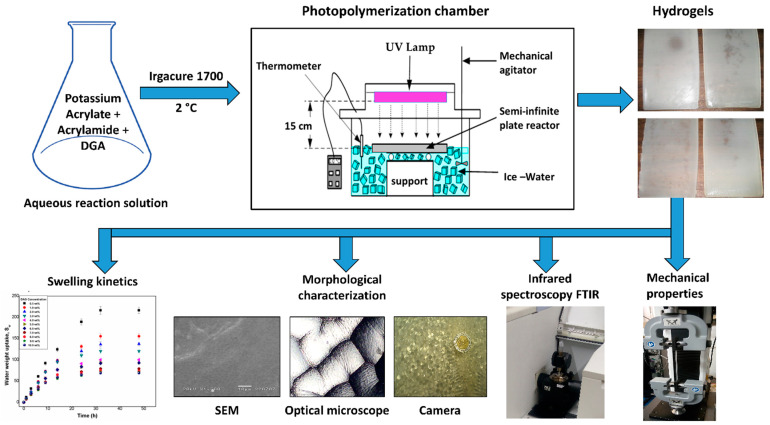
Schematic view of the preparation and characterization of hydrogels.

Figure 9 shows a scheme of the complete methodology of this work. The acrylic acid was dissolved in bidistilled water according to the formulation shown in Table 2. The solution was taken to a pH 7 with the addition of a KOH solution at 47 wt %. The solution was kept below 25 °C to avoid thermal polymerization. Subsequently, the acrylamide and DAG were added, the mixture was stirred until the system was homogeneous, and the temperature was lowered to 2 °C. Finally, 1 mL of photoinitiator solution (3 wt % in methanol) was added. The reaction solution was poured into a 0.450 L glass semi-infinite plate reactor, and nitrogen was bubbled for 15 min. It was placed 15 cm away from the lamp (Tecno F15T8-BLB 20 W, 127 v) rich in 366 nm wavelength radiation, inside an isothermal bath at 2 °C for 1 h (to achieve higher conversion). After the reaction time, the hydrogels were removed, and 0.5 × 0.5 × 0.3 mm^3^ samples were cut and allowed to dry until constant weight, and then were introduced into double-distilled water at 25 °C for three days. The water was changed every 24 h, and they were submerged in the water again for three days at 45 °C. Once the cleaning process was completed, the hydrogels were left to dry at room temperature for 5 days and then put in a vacuum oven at 40 °C until constant weight. The polymer gel fraction (*G_F_*) was calculated as follows:

(1) $$G_{F}\left(crosslinked\;polymer\;\%\right)=\left(\frac{W_{d}}{W_{0}}\right)*100$$

where *W_d_* is the weight of the dry insoluble part of the hydrogel after extraction with water, and *W*_0_ is the initial weight of the xerogel [10].

### 4.3. Swelling Kinetics

Dried samples were weighed and placed in double-distilled water at 25 °C. The samples were taken out and weighed at different times. Absorbent paper was used to remove the excess of water on the surface of samples. Water absorption was calculated by the difference in weight between the weight of the dry sample and the weight of the swollen sample, using the following equation:

(2) $$S_{w}\;\left(\frac{W_{t}-W_{0}}{W_{0}}\right)$$

where *W_t_* and *W*_0_ are the weight of the hydrogel at time *t* and the weight of the xerogel, respectively. To model the swelling kinetics, the second-order model is commonly used, as proposed by Schott [51], which has been used to predict the swelling in acrylic hydrogels [10,20,52]:

(3) $$\frac{dS_{w}}{dt}=K{\left(S_{w\infty }-S_{w}\right)}^{2}$$

where *S_w_* and *S_w∞_* are the swellings at time *t* and at equilibrium swelling, respectively, and *K* is a constant of the system.

Integrating Equation (3), in the limits of *t*, *S_w_*, and *0*, this gives

(4) $$S_{w}=\frac{K\;S_{w}^{2}\;t}{1+K\;S_{w}\;t}$$

Rewriting Equation (4):

(5) $$\frac{t}{S_{w}}=\frac{1}{K\;S_{w\infty }^{2}}+\frac{1}{S_{w\infty }}t$$

Equation (5) represents second-order kinetics. In this case, the rate of swelling at any time is directly proportional to the square of the still available swelling capacity, that is, to the solvent uptake that has not yet occurred before reaching the maximum or equilibrium uptake [52]. 

### 4.4. Morphological Characterization

For morphological characterization, a JEOL JSM 5400 LV scanning electron microscope was used. SEM micrographs were obtained for xerogels, which were coated with gold. The mesostructure of hydrated samples was visualized at different times (10, 20, 30, 40, 40, 60, 120, and 180 s), using an OLIMPUS BX4OF optical microscope with a 40× objective. Images were obtained with a SCC-131A SAMSUNG digital video camera adapted to the microscope. The macrostructure was observed with a Hitachi CCD camera. A coin (21.0 mm of diameter) was used for size comparison.

### 4.5. Mechanical Characterization

Mechanical measurements of the hydrogels were carried out by an SFM-10 Universal Testing Machine. The strain rate was set at 50 mm per minute at room temperature. The experimental data were obtained using an ASTM D-638-14 Standard Test Method [53] as a reference for tensile properties of plastics with eight specimens. Young’s modulus was calculated from the initial slope of the tensile curve.

## Figures and Tables

**Table 1:** Maximum swelling and mechanical tests results for hydrogel samples.

Concentration of DAG (wt %)	Polymer Gel Fraction, *G_F_* (%)	Maximum Swelling	K × 10^8^	Characteristic Length, λ × 10^6^ m	Tensile Strength (Pa)	Young’s Modulus (MPa)	Elongation at Break (%)
0.5	91.2 ± 0.8	216.2 ± 7.5	4.28 ± 0.15	1.46 ± 0.038	1979 ± 19	2.64 ± 0.09	1647 ± 52.4
1.0	92.5 ± 1.1	155.1 ± 5.9	4.72 ± 0.18	1.41± 0.036	2087 ± 17	3.66 ± 0.10	1036 ± 43.1
2.0	92.4 ± 0.9	136.8 ± 4.0	5.62 ± 0.16	1.34 ± 0.039	2121 ± 32	3.73 ± 0.18	871 ± 36.0
3.0	93.6 ± 1.4	120.2 ± 4.3	6.03 ± 0.21	1.18 ± 0.025	2206 ± 16	6.48 ± 0.19	660 ± 34.3
4.0	92.1 ± 0.9	99.7 ± 3.9	5.54 ± 0.22	1.08 ± 0.031	2262 ± 34	7.80 ± 0.22	517 ± 32.0
5.0	93.4 ± 1.2	93.7 ± 3.4	5.93 ± 0.21	1.04 ± 0.016	2261 ± 28	8.84 ± 0.43	391 ± 15.2
6.0	93.1 ± 1.3	93.0 ± 3.5	6.86 ± 0.26	0.99 ± 0.007	2343 ± 24	13.43 ± 0.38	349 ± 23.7
7.0	92.9 ± 1.4	79.2 ± 2.3	8.08 ± 0.23	0.97 ± 0.008	2427 ± 20	14.38 ± 0.86	279 ± 21.0
8.0	91.6 ± 0.9	75.0 ± 2.7	7.38 ± 0.26	0.95 ± 0.005	2435 ± 18	15.25 ± 0.44	251 ± 15.5
9.0	92.0 ± 0.9	70.7 ± 2.8	8.34 ± 0.33	0.92 ± 0.008	2482 ± 26	22.34 ± 1.45	210 ± 19.2
10.0	92.7 ± 1.4	69.4 ± 3.3	10.21 ± 0.49	0.91 ± 0.006	2515 ± 33	28.47 ± 0.81	128 ± 12.5

± standard deviation.

**Table 2:** Formulations of hydrogels obtained.

Substance	Amount (g)										
Acrylamide	49.0										
Acrylic acid	51.0										
DAG	0.5	1.0	2.0	3.0	4.0	5.0	6.0	7.0	8.0	9.0	10.0
Irgacure 1700	0.03										
Water	100.0										
KOH	neutralizer and generating potassium acrylate										

## Data Availability

Data Availability Statement: The data that support the findings of this study are available from the corresponding authors, R.M.J.-A., and S.G.-E., upon reasonable request.
